# Supplementary material for: Ustilago maydis Metabolic Characterization and Growth Quantification with a Genome-Scale Metabolic Model
Source: J Fungi (Basel). 2022 May 20;8(5):524. doi: 10.3390/jof8050524 (PMC9147497; doi:10.3390/jof8050524)
Supplement: Supplementary file 1 [file jof-08-00524-s001.zip › Suppl_7_BiologPlateSubs.pdf]

BIOLOG

Phenotype MicroArrays™

PM1 MicroPlate™ Carbon Sources

A1 Negative Control	A2 L-Arabinose	A3 N-Acetyl-D-Glucosamine	A4 D-Saccharic Acid	A5 Succinic Acid	A6 D-Galactose	A7 L-Aspartic Acid	A8 L-Proline	A9 D-Alanine	A10 D-Trehalose	A11 D-Mannose	A12 Dulcitol
B1 D-Serine	B2 D-Sorbitol	B3 Glycerol	B4 L-Fucose	B5 D-Glucuronic Acid	B6 D-Gluconic Acid	B7 D,L-α-Glycerol-Phosphate	B8 D-Xylose	B9 L-Lactic Acid	B10 Formic Acid	B11 D-Mannitol	B12 L-Glutamic Acid
C1 D-Glucose-6-Phosphate	C2 D-Galactonic Acid-γ-Lactone	C3 D,L-Malic Acid	C4 D-Ribose	C5 Tween 20	C6 L-Rhamnose	C7 D-Fructose	C8 Acetic Acid	C9 α-D-Glucose	C10 Maltose	C11 D-Melibiose	C12 Thymidine
D-1 L-Asparagine	D2 D-Aspartic Acid	D3 D-Glucosaminic Acid	D4 1,2-Propanediol	D5 Tween 40	D6 α-Keto-Glutaric Acid	D7 α-Keto-Butyric Acid	D8 α-Methyl-D-Galactoside	D9 α-D-Lactose	D10 Lactulose	D11 Sucrose	D12 Uridine
E1 L-Glutamine	E2 m-Tartaric Acid	E3 D-Glucose-1-Phosphate	E4 D-Fructose-6-Phosphate	E5 Tween 80	E6 α-Hydroxy Glutaric Acid-γ-Lactone	E7 α-Hydroxy Butyric Acid	E8 β-Methyl-D-Glucoside	E9 Adonitol	E10 Maltotriose	E11 2-Deoxy Adenosine	E12 Adenosine
F1 Glycyl-L-Aspartic Acid	F2 Citric Acid	F3 myo-Inositol	F4 D-Threonine	F5 Fumaric Acid	F6 Bromo Succinic Acid	F7 Propionic Acid	F8 Mucic Acid	F9 Glycolic Acid	F10 Glyoxylic Acid	F11 D-Cellobiose	F12 Inosine
G1 Glycyl-L-Glutamic Acid	G2 Tricarballylic Acid	G3 L-Serine	G4 L-Threonine	G5 L-Alanine	G6 L-Alanyl-Glycine	G7 Acetoacetic Acid	G8 N-Acetyl-β-D-Mannosamine	G9 Mono Methyl Succinate	G10 Methyl Pyruvate	G11 D-Malic Acid	G12 L-Malic Acid
H1 Glycyl-L-Proline	H2 p-Hydroxy Phenyl Acetic Acid	H3 m-Hydroxy Phenyl Acetic Acid	H4 Tyramine	H5 D-Psicose	H6 L-Lyxose	H7 Glucuronamide	H8 Pyruvic Acid	H9 L-Galactonic Acid-γ-Lactone	H10 D-Galacturonic Acid	H11 Phenylethyl-amine	H12 2-Aminoethanol

PM2A MicroPlate™ Carbon Sources

A1 Negative Control	A2 Chondroitin Sulfate C	A3 α-Cyclodextrin	A4 β-Cyclodextrin	A5 γ-Cyclodextrin	A6 Dextrin	A7 Gelatin	A8 Glycogen	A9 Inulin	A10 Laminarin	A11 Mannan	A12 Pectin
B1 N-Acetyl-D-Galactosamine	B2 N-Acetyl-Neuraminic Acid	B3 β-D-Allose	B4 Amygdalin	B5 D-Arabinose	B6 D-Arabitol	B7 L-Arabitol	B8 Arbutin	B9 2-Deoxy-D-Ribose	B10 i-Erythritol	B11 D-Fucose	B12 3-0-β-D-Galactopyranosyl-D-Arabinose
C1 Gentiobiose	C2 L-Glucose	C3 Lactitol	C4 D-Melezitose	C5 Maltitol	C6 α-Methyl-D-Glucoside	C7 β-Methyl-D-Galactoside	C8 3-Methyl Glucose	C9 β-Methyl-D-Glucuronic Acid	C10 α-Methyl-D-Mannoside	C11 β-Methyl-D-Xyloside	C12 Palatinose
D1 D-Raffinose	D2 Salicin	D3 Sedoheptulosan	D4 L-Sorbose	D5 Stachyose	D6 D-Tagatose	D7 Turanose	D8 Xylitol	D9 N-Acetyl-D-Glucosaminitol	D10 γ-Amino Butyric Acid	D11 δ-Amino Valeric Acid	D12 Butyric Acid
E1 Capric Acid	E2 Caproic Acid	E3 Citraconic Acid	E4 Citramalic Acid	E5 D-Glucosamine	E6 2-Hydroxy Benzoic Acid	E7 4-Hydroxy Benzoic Acid	E8 β-Hydroxy Butyric Acid	E9 Glycolic Acid	E10 α-Keto-Valeric Acid	E11 Itaconic Acid	E12 5-Keto-D-Gluconic Acid
F1 D-Lactic Acid Methyl Ester	F2 Malonic Acid	F3 Melibionic Acid	F4 Oxalic Acid	F5 Oxalomalic Acid	F6 Quinic Acid	F7 D-Ribono-1,4-Lactone	F8 Sebacic Acid	F9 Sorbic Acid	F10 Succinamic Acid	F11 D-Tartaric Acid	F12 L-Tartaric Acid
G1 Acetamide	G2 L-Alaninamide	G3 N-Acetyl-L-Glutamic Acid	G4 L-Arginine	G5 Glycine	G6 L-Histidine	G7 L-Homoserine	G8 Hydroxy-LProline	G9 L-Isoleucine	G10 L-Leucine	G11 L-Lysine	G12 L-Methionine
H1 L-Ornithine	H2 L-Phenylalanine	H3 L-Pyroglutamic Acid	H4 L-Valine	H5 D,L-Carnitine	H6 Sec-Butylamine	H7 D,L-Octopamine	H8 Putrescine	H9 Dihydroxy Acetone	H10 2,3-Butanediol	H11 2,3-Butanedione	H12 3-Hydroxy-2-Butanone
